# Soft Clipping: An Often-Overlooked Non-Linear Amplitude Response of IEPE/ICP Piezoelectric Sensors

**DOI:** 10.3390/s26154999

**Published:** 2026-08-06

**Authors:** Oliver M. Zobel, Johannes Maierhofer, Michael Kreutz, Daniel J. Rixen

**Affiliations:** Chair of Applied Mechanics, TUM School of Engineering and Design, Technical University of Munich, Boltzmannstr. 15, 85748 Garching, Germany

**Keywords:** sensors, integrated electronics, acquisition hardware, operational vibrations, experimental dynamics

## Abstract

This article reviews soft clipping, a non-linear amplitude response of IEPE/ICP sensors, which occurs when the internal electronics of an IEPE/ICP sensor exceed their specified linear operating range, typically corresponding to an output of ±5 V. Because most measurement systems allow a wider voltage range of ±10 V, this non-linear behavior can occur without triggering system-level warnings. Shaker tests indicate that prolonged operation beyond the rated measurement range results in significant signal distortion, particularly attenuating negative acceleration values and producing asymmetric signals. Even after returning to nominal operating conditions, a recovery time of several seconds is required. While the soft clipping effect appears largely frequency independent up to 5 kHz, it may be difficult to detect in real-world vibration tests with complex, broadband signals. Impact tests further reveal that soft clipping subtly affects shock responses, primarily altering initial peaks and potentially obscuring high-frequency modes that decay rapidly. In contrast, frequency-domain analyses are less affected. Overall, the findings emphasize that avoiding operation outside the rated measurement range and implementing appropriate monitoring or warning strategies are essential for reliable IEPE/ICP measurements, especially for time-domain analysis methods.

## 1. Introduction

Piezoelectric accelerometers with integrated electronics (IEPE, also referred to with the proprietary terms ICP®, Isotron®, Deltatron®, Piezotron® or CCLD [1]) are the de facto standard for acceleration measurements. Compared to older piezoelectric acceleration sensors, IEPE sensors already include the required integral electronics to convert the generated charges to a measurable voltage. There are many reasons to include the required electronics in the sensor, instead of relying on external charge amplifiers. These include ease of use, reduced susceptibility to external electrical disturbances and ground-loop issues, as well as an increased dynamic range [2].

**Figure 1:**
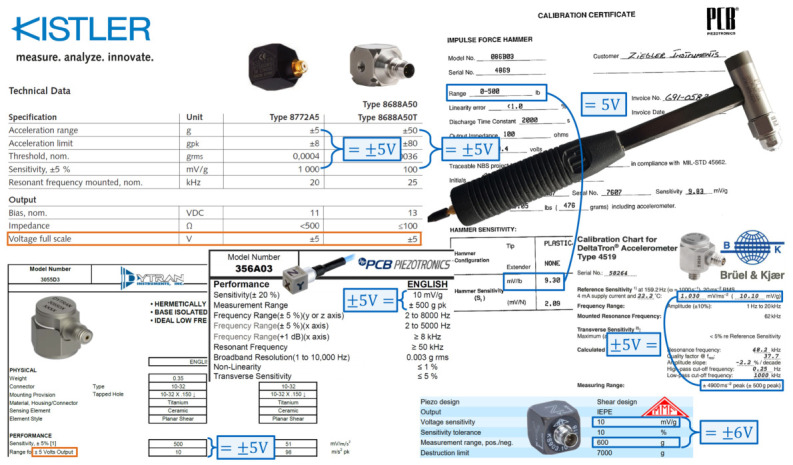
Excerpts from various IEPE sensors, showing the limited measurement range of commonly ±5 V. This can be determined from the measurement range and the sensitivity range (marked in blue) and is only sometimes directly noted in the datasheets (marked in orange).

However, integrating the charge amplifier into the sensor also has some drawbacks. Besides the inherently fixed amplification factor, which requires multiple sensors to cover different measurement ranges and sometimes means buying three or four different sensors instead of one. There is another, non-obvious drawback: for most IEPE sensors, the linear voltage output scale is limited to ±5 V [2]. While this limited voltage output range is documented in the sensor’s datasheet, this is often done only indirectly. Figure 1 shows excerpts of sensor datasheets from *Kistler*, *PCB Piezotronics*, *Dytran Instruments*, *Brüel & Kjær*, and *MMF*. As can be seen, only the datasheets of the *Dytran 3055D3* sensor and the two *Kistler* sensors directly specify the voltage range of ±5 V. For the other sensors, this is given implicitly through the maximum measurement range, e.g., an acceleration range of ±500 g, multiplied by the sensitivity, e.g., 10 mV/g, which equals a voltage range of ±5 V. Some sensors, however, can have a different voltage range, e.g., the *MMF KS903B100* has a voltage range of ±6 V, other sensors might have even higher ranges.

A problem arises in practice because the sensor’s voltage range does not coincide with the common voltage input range of ±10 V found for many commercial acquisition systems. This mismatch can lead to a saturation effect that is not only present for continuous loads, such as during shaker testing or operational vibration measurements but also for transient events like impacts, as is shown in this article. This saturation, or also referred to here as soft clipping, deteriorates the signal in a non-linear and non-obvious way. Since usually there is no overload indication for this, when the measurement range is set to ±10 V and no other limit is set up, this can lead to hard-to-spot issues.

A practical example of this issue arose during validation tests of *OASIS-UROS* [3], the latest *Open Acquisition System for IEPE Sensors* developed by the authors, prompting a detailed investigation of the IEPE technology that motivated this article.

**Figure 2:**
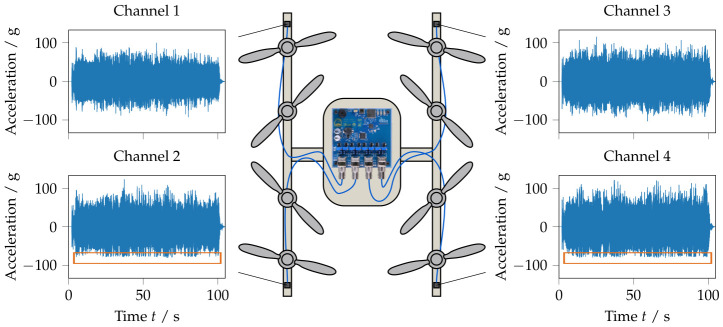
Sketch of octocopter model test with four accelerometers and an *OASIS-UROS* board. The time series of the measured accelerations is shown next to the respective sensor.

The system under test was an octocopter-shaped prototype, see Figure 2, on which four acceleration sensors, two with ±50 g and two with ±500 g range, and an *OASIS-UROS* board were mounted. The voltage ranges of the acquisition system were set to ±12.5 V. With the available eight channels, the accelerations at the four ends of the blade mounting bars were measured in vertical and lateral directions. The four operational accelerations measured in the vertical direction, i.e., perpendicular to the drawing plane, are also shown in Figure 2 next to the respective sensor.

At first glance, the measured time data appear to be as expected; however, upon closer examination of the negative accelerations in channels 2 and 4, it seems that the measured signal is clipped. Moreover, the oscillations are not symmetric with respect to the horizontal axis. When the raw data are inspected, it can be seen clearly that the ±5 V range of the sensor is exceeded. This example demonstrates that when the sensor’s measurement range of ±5 V is greatly exceeded, the signal is heavily distorted.

Nevertheless, there are still some questions regarding what happens when the measurement range is exceeded: What exactly causes the soft clipping? How bad is this for the measurements? Is it possible to detect soft clipping after the measurements have been converted to mechanical quantities or transformed into the frequency domain? What if the measurement range is only slightly exceeded or only for a short time?

While the datasheets would suggest avoiding leaving the measurement range altogether, in practical applications it is still beneficial to know exactly what is happening and how the measurements might be negatively influenced. This allows for an informed decision, e.g., whether a measurement that peaked at 5.2 V should be discarded entirely or whether the signal distortion remains negligible.

The goal of this article is to explain the saturation effect, referred to as soft clipping in the following, based on the working principle of the IEPE circuitry, so that test technicians and mechanical engineers can understand it. It also provides practical examples where this issue occurs and how it can be recognized. Those examples focus on accelerometers; however, the same discussion applies to other IEPE transducers, such as force sensors.

It is difficult to judge whether soft clipping has affected existing publications due to the non-linearity and the transient recovery process after an IEPE sensor is driven outside its measurement range. Without a known-good reference, it is very difficult to determine whether the signal’s amplitude was distorted by soft clipping, especially when operational vibrations are measured, where the signal content is unpredictable.

This contribution first explains the working principle of the IEPE circuitry. Then, it details the reason for soft clipping, the non-linear amplitude response of IEPE sensors outside their rated measurement range. Then, the practical relevance of the limited output range of commonly ±5 V, as stated in many IEPE sensor datasheets, is investigated through laboratory experiments under controlled conditions. First, the behavior is analyzed for operational vibration measurements, simulated with shakers. Second, the behavior during shocks is emulated using impact testing. Lastly, based on the results, the practical relevance of the discussed limitations is analyzed.

## 2. Working Principle of the IEPE Circuitry and Soft Clipping

In this section, the fundamental working principle of the electronics utilized in IEPE sensors is explained. These electronics are required to convert the high-impedance charge output of piezoelectric sensors into a low-impedance voltage source that can be measured by acquisition systems. Then, the soft clipping issue of the integrated IEPE circuit is detailed.

### 2.1. Working Principle of the IEPE Circuitry

**Figure 3:**
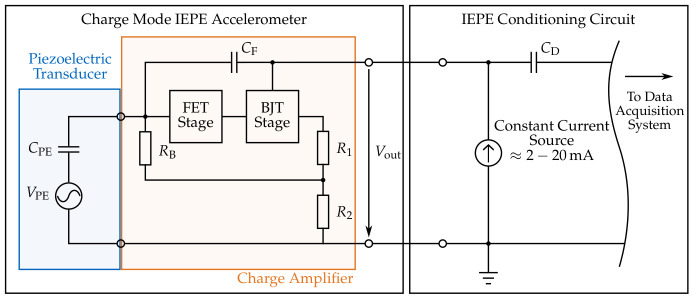
Schematic overview of a charge-mode IEPE accelerometer, consisting of a piezoelectric transducer and charge amplifier, connected to an IEPE conditioning circuit, based on [2].

For IEPE sensors, the charge amplifier is directly integrated into the sensor. A schematic overview of an IEPE sensor attached to the required IEPE conditioning circuit can be seen in Figure 3. While the integrated charge amplifier could also be realized using an op amp (with at least three cables), this is commonly not done. Instead, a combination of a FET (Field-Effect Transistor) input stage with a BJT (Bipolar Junction Transistor) output stage is used. Compared to an op-amp-based circuit, a FET-BJT amplifier offers a simpler and smaller footprint, a lower noise floor, higher operating temperatures, and allows for two-wire operation, eliminating the need for additional power supply cables [2].

Another benefit of IEPE sensors is the easy integration of TEDS (Transducer Electronic Data Sheet). On a small EEPROM (Electrically Erasable Programmable Read-Only Memory) chip, commonly a *DS2430A* or *DS2431* chip, the manufacturer can store information about the IEPE sensor, such as the calibrated sensitivity, sensor type, and serial number. TEDS are generally standardized in IEEE 1451 [4], while the standard IEEE 1451.4 [5] specifically addresses the integration into IEPE sensors. By using diodes and a negative voltage, TEDS can be read over the two-wire connection of IEPE sensors [2,6].

The power supply of IEPE sensors is realized with a constant current source that provides 2–20 mA. There is no general standard for the supplied current and the actually supplied value may differ between data acquisition systems [2,7,8]. However, the value of the supply current influences the high-frequency response and the maximum frequency that can be transmitted. Increasing the supply current for a given cable capacitance or length increases that limit [7,9,10]. Furthermore, the constant current source requires a compliance voltage of 22–30 V [2,7,8].

The feedback capacitor $C_{F}$ can be used to adjust the charge gain of the amplifier. The biasing resistor $R_{B}$ is used to bias the FET, while the voltage divider formed by $R_{1}$ and $R_{2}$ is used to adjust the so-called bias voltage of the sensor [2].

**Figure 4:**
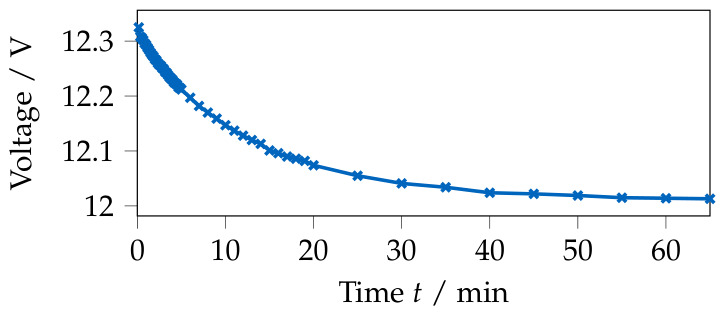
Measurement of an IEPE sensor’s output voltage using a multimeter without a high-pass filter, showing the drift of the bias voltage over time.

When a constant current source is connected to an IEPE sensor, a DC voltage, the bias voltage, can be measured at the sensor’s output. The bias voltage is different for each sensor, depends on the internal circuit and temperature, and will drift over time [2,7,8]. An exemplary measurement of the bias voltage drift over time can be seen in Figure 4. This constant voltage is commonly removed by the IEPE conditioning circuit using a high-pass filter, depicted in Figure 3 with the decoupling capacitor $C_{D}$ [2,7,8].

**Figure 5:**
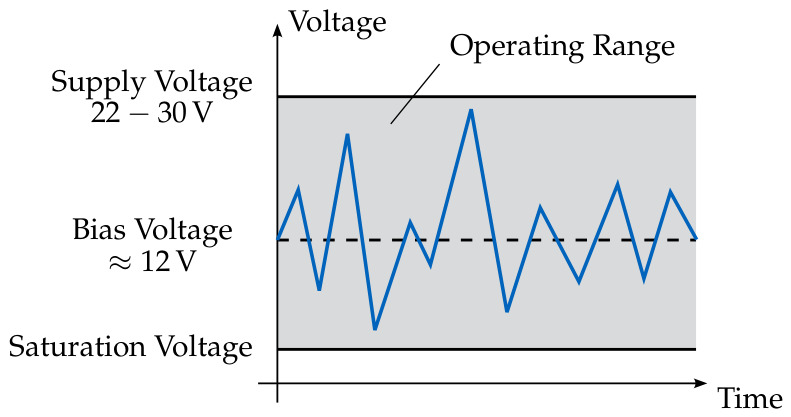
Voltage range of an IEPE sensor, based on [2,7,8].

The measurement signal of the sensor, e.g., the acceleration that is measured, is imposed on the bias voltage, as shown in Figure 5. In other words, the accelerations to be measured are represented as a voltage signal oscillating around the bias voltage [2,7,8].

The operating range of an IEPE sensor, i.e., the voltages that the IEPE circuitry can realize, are limited at the top and the bottom. On the upper end, the sensor is limited by the supply voltage, which cannot be exceeded. On the lower end, the sensor can never reach 0 V with an active IEPE supply, due to the voltage divider formed by $R_{1}$ and $R_{2}$, and the voltage cannot become negative [2,7,8]. However, there is another limit on the lower end, represented by the saturation voltage of the amplifier circuit [7,8].

### 2.2. Soft Clipping of IEPE Sensors

**Figure 6:**
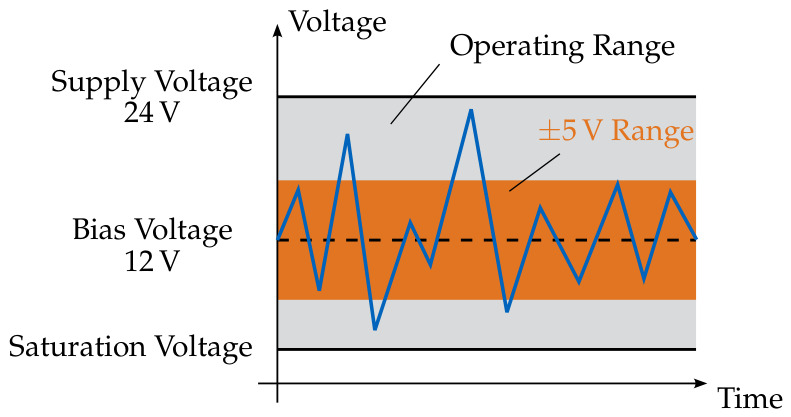
Voltage range of an IEPE sensor with marked ±5 V range, based on [2,7,8].

As was discussed in the introduction, most IEPE sensors’ output is limited to a range of commonly ±5 V (see also Figure 1), i.e., a voltage of ±5 V around the bias voltage. Since the highest (AC-coupled) voltage range of data acquisition systems is often around 10 V, there is no overload warning when the ±5 V range is exceeded. Further, when adding the ±5 V range to Figure 5, see Figure 6, it can be seen that a sensor just barely exceeding the ±5 V range is still far away from the hard voltage limits, i.e., the point where a hard clipping would occur. The question is, what happens in the area between the ±5 V range and the hard limits?

The limitation of IEPE sensors outside of the ±5 V range is introduced by the integrated electronics. While the electronics do not stop functioning outside of the specified voltage range, their behavior becomes non-linear [2]. Even inside the measurement range, there could be some slight amplitude non-linearity, depending on the sensor manufacturer and model. For example, both the *Kistler 8688A50T* and *Dytran 3055D3* sensors have an amplitude non-linearity of ±1% within the rated measurement range [11,12].

The reason for soft clipping is most likely saturation of either the FET or BJT stage in the charge amplifier. A BJT, for example, can show saturation effects when its base current becomes too high. It can then no longer provide its rated amplification factor β, i.e., the relation between base current $i_{B}$ and collector current $i_{C}$ is no longer the linear relation $i_{C}=\beta i_{B}$ [13].

## 3. Operational Vibrations—Shaker Test

First, the behavior of IEPE sensors exceeding their ±5 V measurement range, here referred to as soft clipping, is analyzed for operational vibrations, simulated using a shaker. This means the system under test is constantly excited, here by the shaker, i.e., a forced response is analyzed. This section serves as an initial, controlled reproduction of the soft clipping phenomenon in the lab. First, the behavior in the time domain is evaluated using a fixed-frequency sine excitation with varying amplitude. Second, the frequency dependence is investigated using a controlled stepped sine test.

### 3.1. Time Responses of Sensors Gradually Driven in Overload

After describing the test setup, two cases are investigated: a slow increase in the sine’s amplitude from zero to a value where the physical measurement range of one sensor is exceeded (soft clipping), but the voltage range of the measurement system is not exceeded. And a quick, but continuous, jump from a regular operation to an overload state, and vice versa.

#### 3.1.1. Test Setup

**Figure 7:**
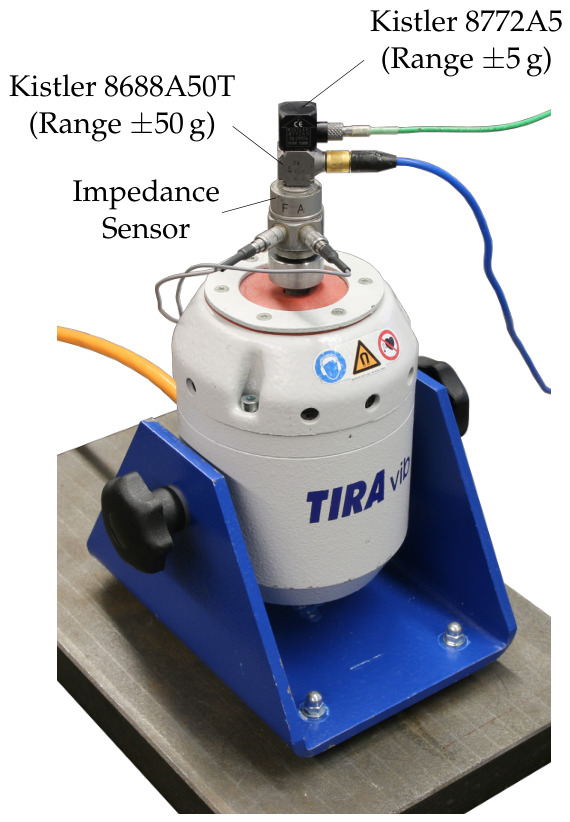
Shaker test setup with two different accelerometers with ±5 g and ±50 g range.

An overview of the used shaker test setup is given in Figure 7. On a *TIRAvib S 50018* shaker, a *Dytran 5860B* impedance sensor was fixed using a threaded adapter. On top of it, a *Kistler 8688A50T* triaxial acceleration sensor was mounted with a threaded insert. Both sensors have a physical measurement range of ±50 g (corresponding to ±5 V) according to their datasheets [11,14]. On the top of the ±50 g sensor, a *Kistler 8772A5* uniaxial acceleration sensor was glued. As per its datasheet [15], the measurement range is only ±5 g.

An *LMS SCADAS mobile measurement system* was used to record the time data of all three sensors with a sample rate of 204.8 kHz. This high sample rate was chosen to be able to clearly see changes in the amplitudes in the raw time series. The shaker was driven through either the *LMS SCADAS mobile measurement system* for the results in Section 3.1.2 or a *RIGOL DG1032Z* signal generator for the results in Section 3.1.3. For both cases, a sine wave with a fixed frequency of 100 Hz or 1 kHz was used.

#### 3.1.2. Slow Increase of Amplitude

**Figure 8:**
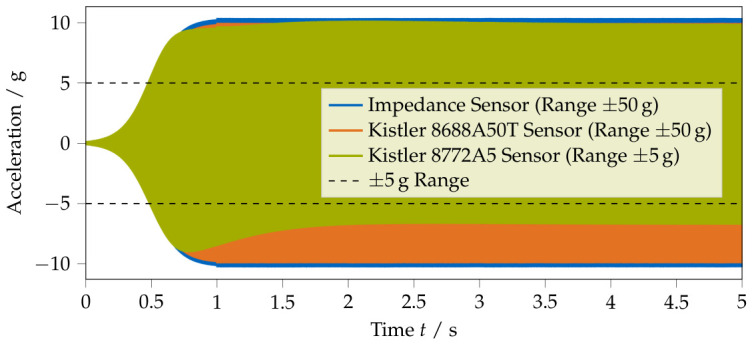
Time series of measured accelerations for excitation with a 1 kHz sine, and amplitude gradually increased to approximately 10 g (measured by the impedance sensor) over 1 s. The dashed lines indicate the ±5 g measurement range of the *Kistler 8772A5* sensor according to its datasheet.

For the first test, the amplitude of the sine wave was increased continuously from zero to an acceleration amplitude of approximately 10 g over a time of 1 s. The measured acceleration time series for an excitation with a 1 kHZ sine wave can be seen in Figure 8. The results for a 100 Hz sine wave are qualitatively identical.

Both the impedance sensor and the ±50 g sensor give the expected result: between t=0 and t=1 s, the acceleration amplitudes increase continuously and reach the set value of ≈10 g. For the ±5 g sensor, however, a different behavior can be observed: starting at t≈0.7 s, the magnitude of the negative acceleration peaks stops increasing and then even decreases until t≈2 s. Looking at the positive acceleration peaks, it can be observed that the amplitudes of the ±5 g sensor slightly increase after t=1 s. Looking at the accelerations measured by the ±5 g sensor after t=2 s, it can be seen that the oscillation is not symmetric with respect to the horizontal axis, i.e., the acceleration is not around zero. This is the effect referred to as soft clipping.

This is the same behavior as seen with the introduction example; however, here, the issue is significantly easier to spot, especially since the measurement range of the ±5 g sensor was exceeded by 100%. Without any doubt, such a measurement, where the acceleration amplitudes constantly and significantly exceed the sensor’s measurement range, are useless. Nevertheless, the remaining practical issue is that for the measurement shown, no overload indication was given by the measurement system, since the used voltage range of ±12 V was never exceeded. Especially for operational vibration measurements, where potentially only the calculated spectra are saved and viewable, this poses a serious problem.

**Figure 9:**
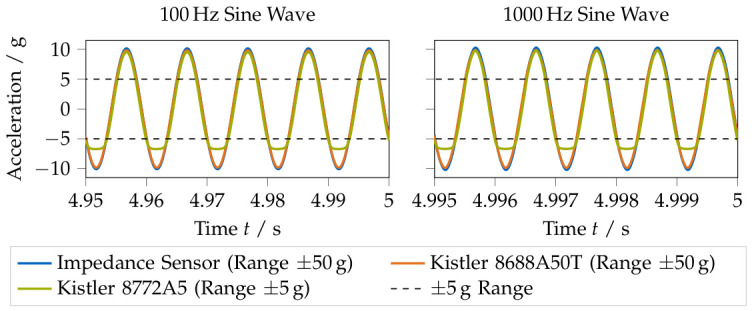
Close-up of 100 Hz and 1 kHz sine wave time series toward the end of the tests. The dashed lines indicate the ±5 g measurement range of the *Kistler 8772A5* sensor according to its datasheet.

A close-up of the sine waves toward the end of the measurement, i.e., after the ±5 g sensor has settled, is shown in Figure 9 for both the test with a 1 kHz sine wave and with a 100 Hz sine wave. As can be seen for both frequencies, the positive peaks of the ±5 g sensor pretty closely match the sine wave measured by the impedance sensor and the ±50 g sensor. However, for the negative peaks, it can be clearly seen that the sine wave is heavily distorted. At the lowest point, the ±5 g sensor measures ≈−6.7 g. Assuming the bias voltage to be 11.8 V and the sensitivity to be 978 mV/g (according to the sensor’s calibration), this means the lowest DC-coupled voltage was about 5.25 V.

**Figure 10:**
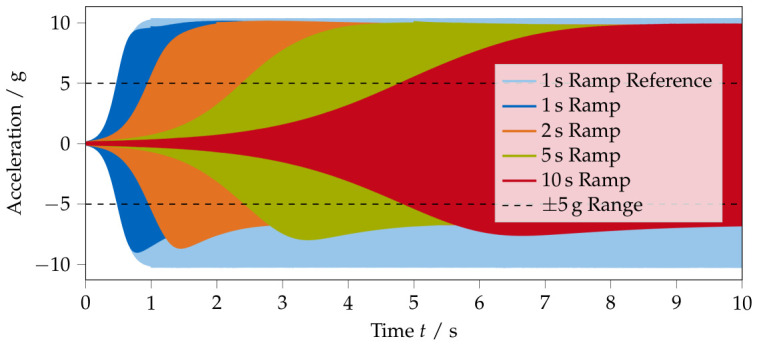
Time series of measured accelerations for excitation with a 1 kHz sine. The acceleration amplitude is gradually increased to approximately 10 g (measured by the impedance sensor) over a period of 1, 2, 5, and 10 s. As a reference, the measurement of the impedance sensor with a ramp time of 1 s is given. The dashed lines indicate the ±5 g measurement range of the *Kistler 8772A5* sensor according to its datasheet.

In Figure 10, the same test is shown for a ramp time of the sine wave excitation of 1, 2, 5, and 10 s. Here, only the results of the ±5 g sensor is shown, along with a reference measurement of the impedance sensor for a ramp time of 1 s. Compared to the already discussed results for a 1 s ramp time, the other ramp time results show qualitatively the same behavior: at some point, the magnitude of the negative acceleration peaks is decreasing. After some settling time, a vibration with constant amplitude is measured; however, this oscillation is not symmetric.

When comparing the different ramp times, it can be seen that the soft clipping, or the saturation of the IEPE circuit, does not occur immediately but depends on time. This can be seen by observing that for a ramp time of 1 s, a higher magnitude of the negative acceleration peaks is achieved, as compared to a 5 s ramp time. If only the magnitude of the overload matters, the same values of the negative acceleration peaks should be found for each ramp time. Since this is not the case, the behavior of IEPE sensors during soft clipping must also depend on time. This might imply that soft clipping is less of an issue for shock responses, where potentially only a few vibration cycles exceed the measurement range. The temporal behavior for continuously excited vibrations was further explored with the next test.

#### 3.1.3. Quick Jump in Amplitude

**Figure 11:**
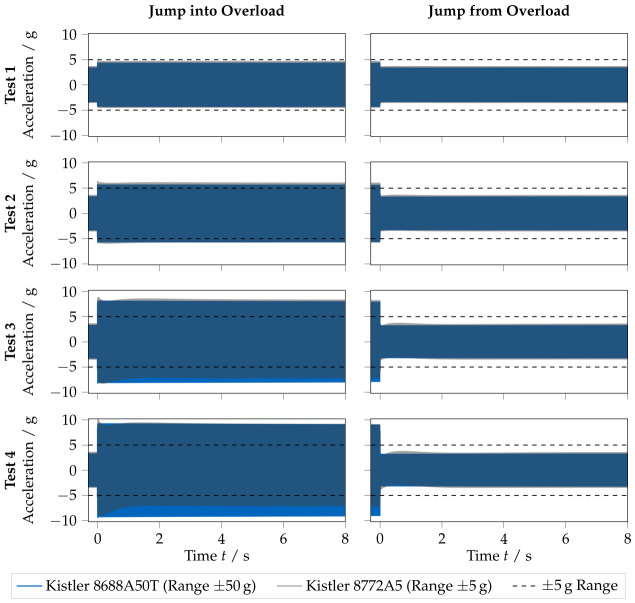
Time series of measured accelerations by the two sensors excited with a 100 Hz sine wave. The shaker is operated in a steady state before t=0, and then at t=0, the amplitude is quickly and continuously increased (plots on the left) or decreased (plots on the right). This test is performed at four different acceleration levels, ranging from slightly under 5 g (no overload) in the top plot to as close to 10 g as possible in the bottom plot. To enable simultaneous viewing of both sensors’ measurements, the **lines of the *Kistler 8688A50T* sensor are plotted partially transparent**. The dashed lines indicate the ±5 g measurement range of the *Kistler 8772A5* sensor according to its datasheet.

Next, the behavior of IEPE sensors for quick, but continuous, amplitude jumps was investigated. For this, two variants were performed for each test: first, a ‘Jump into Overload’, where all sensors were first operated inside their measurement range and then the amplitude was increased such that the ±5 g sensor exceeded its measurement range; and second, a ‘Jump from Overload’, where the ±5 g sensor was operated in overload and then returned to its measurement range. This test was performed with a 100 Hz and a 1 kHz sine wave, and for four different degrees of overload, denoted as ‘Test 1’ to ‘Test 4’ in the following. The test results using a 100 Hz sine wave are shown in Figure 11; the results using a 1 kHz sine wave are qualitatively identical.

For ‘Test 1 - Jump into Overload’, the jump in amplitude at t=0 is to a level close to, but not exceeding, the 5 g limit. As can be seen, besides a small difference in amplitude, the ±5 g and ±50 g sensors basically give the same measurement. However, for the ±5 g sensor, a slight asymmetry of the oscillations after t=0 can already be seen, even though the measurement range is not yet exceeded. The same can be observed for ‘Test 1 - Jump from Overload’.

When the measurement range is exceeded, as is the case for ‘Test 2’ through ‘Test 4’, an additional phenomenon can be observed: after the ‘Jump into Overload’, the ±5 g sensor overshoots (compared to the ±50 g sensor) and then settles for a few seconds. During this, the magnitudes of the negative accelerations are being attenuated. Note that the lines of the ±50 g sensor are plotted partially transparent. This means for dark blue areas that the plot of the ±5 g sensor’s measurement is behind the ±50 g sensor plot. The same settling behavior can be seen for the ‘Jump from Overload’ for ‘Test 2’ to ‘Test 4’, where the ±5 g sensor also needs a few seconds to settle.

This test shows that when the measurement range of an IEPE sensor is continuously exceeded, as, for example, in operational vibration measurement, some time is required for the IEPE circuitry to recover.

The recovery time after an overload, i.e., when the sensor is driven outside its operating range, depends on the time constant of the sensor’s amplifier [16] and varies across sensor models and manufacturers. For recovery to begin, the sensor must be returned to its rated operating range [16].

In real-world operational measurements, a saturated amplifier might introduce only subtle signal distortions that are hard to identify in the measured data, which is often already converted to physical units. Therefore, leaving the rated measurement range should be best avoided, and this should already be checked during the measurement.

### 3.2. Frequency Response with Controlled Amplitude Stepped Sine

To conclude the shaker tests, the soft clipping phenomenon was investigated in the frequency domain using a stepped-sine test with controlled amplitudes. The goal was to investigate whether the amplitude attenuation was dependent on the vibration frequency.

#### 3.2.1. Test Setup

**Figure 12:**
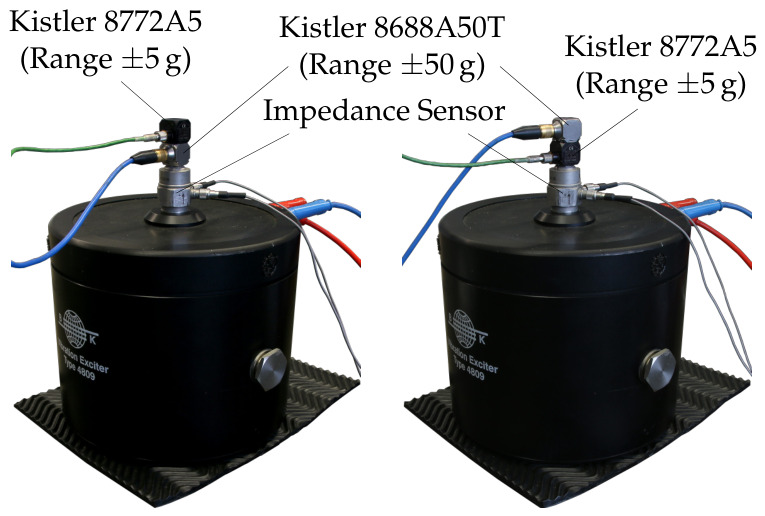
Second shaker test setup for stepped sine tests with two different accelerometers with ±5 g and ±50 g range. Between tests, the sensor stack-up was swapped.

For the tests, the shaker setup shown in Figure 12 was used. On a *Brüel & Kjær Type 4809* shaker, a *Dytran 5860B* (datasheet [14]) impedance sensor was fixed with a threaded adapter. Attached to it were a *Kistler 8688A50T* (datasheet [11]) triaxial acceleration sensor and a *Kistler 8772A5* (datasheet [15]) uniaxial acceleration sensor. The idea of the vertical sensor stack is that both accelerometers are subjected to the same acceleration; however, for higher excitation frequencies, this is not the case. To account for that, two tests were performed, one where the ±5 g sensor was on top and one where the ±50 g sensor was on top.

An *LMS SCADAS mobile measurement system* was used for the controlled stepped sine test. First, a system identification (FRFs) was performed using a pseudo-random excitation, which was then used by the *LMS* to control the shaker such that an acceleration level of 2, 4, 6, 8, and 10 g was achieved for the impedance sensor in a frequency range of 100 to 5000 Hz.

#### 3.2.2. Stepped Sine Results

**Figure 13:**
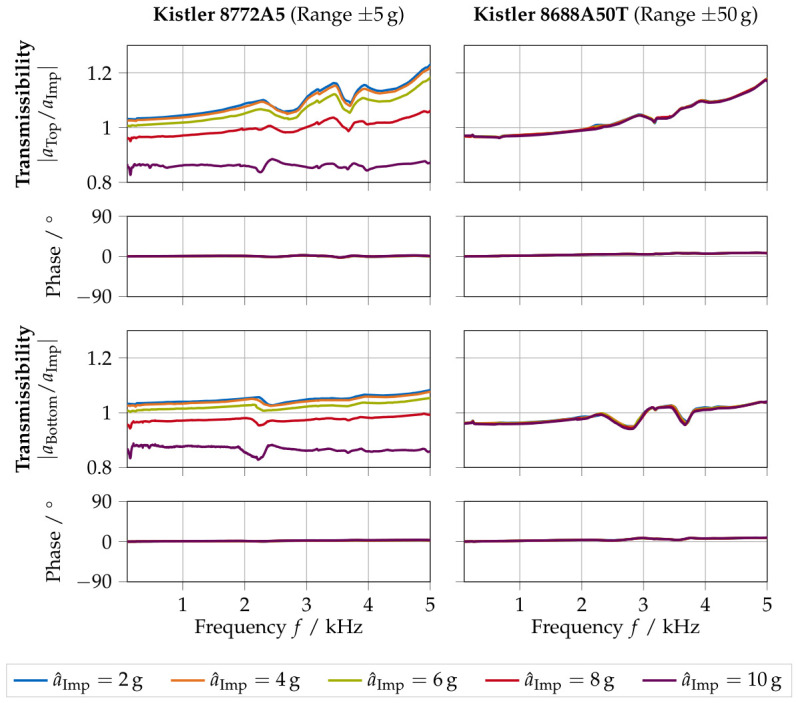
Results of the controlled amplitude stepped sine for two sensor-stack configurations as shown in Figure 12. Shown are the magnitude and phase of the transmissibility between the sensor under test and the impedance sensor.

The results of the stepped sine test are shown in Figure 13 in the form of transmissibilities, i.e., transfer functions between the sensor under test and the impedance sensor, for the five different acceleration amplitudes ${\hat{a}}_{\mathrm{Imp}}$ of the impedance sensor. On the left, the results for the ±5 g sensor are shown, and on the right, the results for the ±50 g sensor. The upper plots belong to the configurations where the respective sensor was on top of the sensor stack, the lower plots where the sensor was at the bottom. This means that the transmissibility shown in the top left and bottom right, respectively, top right and bottom left, corresponds to the same physical configuration.

As can be seen, the acceleration magnitude of the sensors in the top position increases significantly for higher frequencies. At 5 kHz, all sensors are within their rated frequency bandwidth and far away from their eigenfrequency. The increased amplitudes for the top sensors are probably not caused by picked-up transverse vibrations because the sensor’s transverse sensitivity is rated as <5%. More likely is that the connection between the bottom and top sensor is not entirely rigid at the higher frequencies.

For the ±50 g sensor, it can be seen that in both configurations, the transmissibility is not affected by a different acceleration magnitude. The curves of the five different impedance sensor acceleration amplitudes ${\hat{a}}_{\mathrm{Imp}}$ coincide. This is, however, not the case for the ±5 g sensor. The difference in magnitude between the curves for ${\hat{a}}_{\mathrm{Imp}}=2g$ and ${\hat{a}}_{\mathrm{Imp}}=4g$ is already bigger than the difference between any curves for the ±50 g sensor. For the ±5 g sensor in the top position and ${\hat{a}}_{\mathrm{Imp}}=4g$, the acceleration amplitude measured by the ±5 g sensor is already close to 5 g, i.e., the sensor is already operating at the edge of its rated acceleration measurement range.

At higher acceleration levels ${\hat{a}}_{\mathrm{Imp}}$, it can clearly be seen that the acceleration magnitude measured by the ±5 g sensor is decreasing. This decrease appears to be mostly independent of the excitation frequency, at least for the frequency ranges used here; note that the transmissibility is plotted on a linear scale.

For continuously driven systems, it has been shown that the amplitude attenuation due to soft clipping is relatively independent of frequency; however, whether leaving the measurement range for only a short time, e.g., during impact tests, is detrimental to the measurement has not yet been investigated.

## 4. Shock Responses—Impact Tests

In this section, the soft clipping effect is analyzed for shock responses using impact testing. For this, two test cases are analyzed: first, impact testing is performed using a manual impact hammer on an aluminum structure suspended by rubber ropes. This test utilizes the same sensors used in the previous shaker tests. While both sensors are from the same manufacturer, the internal construction is likely to be different, as one sensor is a uniaxial accelerometer, while the other is a triaxial accelerometer. Therefore, a second impact test is performed with sensors of the same type but with different sensitivities. That test is performed using an automatic impact hammer on a clamped aluminum plate.

### 4.1. Aluminum Structure Hanging on Rubber Ropes

The first impact test is performed on an aluminum structure normally used for substructuring benchmarks with approximated ‘free–free’ boundary conditions. After a description of the test setup, two tests are evaluated: first, the time responses to impacts with peak acceleration amplitudes close to 10 g, two times the rated measurement range of the used ±5 g sensor, are analyzed. Second, frequency response functions are measured with varying impact force, resulting in acceleration responses within and outside the ±5 g sensor’s measurement range.

#### 4.1.1. Test Setup

**Figure 14:**
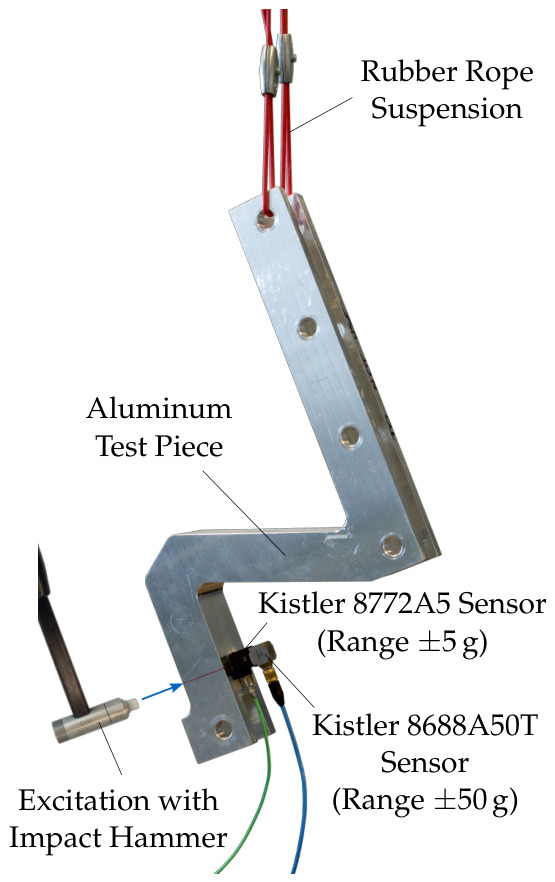
Impact test setup with two different *Kistler* accelerometers with acceleration ranges of ±5 g and ±50 g.

An overview of the test setup is given in Figure 14. The aluminum structure was impacted with an impact hammer with a vinyl tip, and on the other side of the solid aluminum section, two sensors were attached using glue. As for the shaker test, a *Kistler 8688A50T* (datasheet [11]) triaxial acceleration sensor with a ±50 g range and a *Kistler 8772A5* (datasheet [15]) uniaxial acceleration sensor with a ±5 g range were used. A *Müller-BBM PAK MKII* system was used for data acquisition with a sample rate of 102.4 kHz.

For this test, the vertical stacking order and the orientation of the sensors were varied. While the idea is that two sensors glued on top of each other measure the same vibration, in reality, this is not the case. Changing the stacking order allowed us to judge how good the assumption really was. The orientation of the sensors was also changed because it was observed with the shaker test that the soft clipping mostly attenuated the negative accelerations, i.e., accelerations that corresponded to a DC-coupled sensor voltage smaller than the bias voltage.

#### 4.1.2. Time Responses to Impacts for Different Sensor Configurations

**Figure 15:**
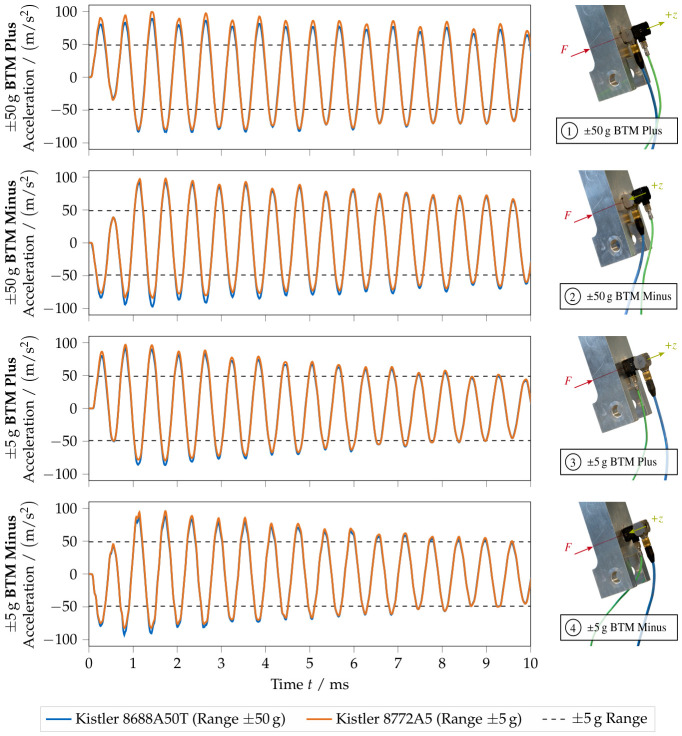
Impact testing results for the four sensor configurations described above and depicted next to the matching plot. The figure shows the initial shock response over time for the *Kistler 8688A50T* sensor (always within measurement range) and the *Kistler 8772A5* sensor, which is in overload outside of the ±5 g range as indicated by the dashed lines.

The initial time responses to the impacts for the four different configurations are shown in Figure 15. For the configurations where the first acceleration response peak is positive, i.e., for configurations ① and ③, the positive response magnitudes for the ±5 g sensor are actually higher than those for the ±50 g sensor, which seems counterintuitive at first; however, this is in line with the observations for the shaker test with an instantaneous jump in amplitude (Figure 11), where, immediately after the jump in amplitude, the positive magnitudes were also higher than those for the ±50 g sensor. The attenuation of the negative acceleration magnitudes only slowly developed after that.

For configurations ① and ③, a decrease in the negative acceleration peaks can also be observed; this is more clearly visible for configuration ③. Additionally, it can be observed that the response time series differ slightly in terms of decay behavior, depending on which sensor is mounted at the bottom. At the end of the 10 ms measurement, the response peaks for configurations ①; and ②; are still outside of the ±5 g range, while for ③ and ④, the peaks lie within the ±5 g range. At the end, both sensors measure basically the same amplitudes, except for configuration ①, where the ±5 g sensor still shows slightly higher amplitudes.

For configurations ② and ④, the positive response magnitudes are only slightly higher for the ±5 g sensor, while the negative peaks are clearly attenuated. While it appears that the ±5 g sensor has fully recovered from soft clipping after 10 ms, especially for configurations ③ and ④, the initial response peaks are clearly wrong. Despite the sensor only shortly leaving its measurement range, the measurement might already be compromised, depending on the subsequent processing method.

This test shows that the soft clipping effect can significantly distort the initial shock response. Without a reference, which usually does not exist, this error will be difficult to spot in measurements.

#### 4.1.3. Frequency Response Functions for Varying Degree of Overload

**Figure 16:**
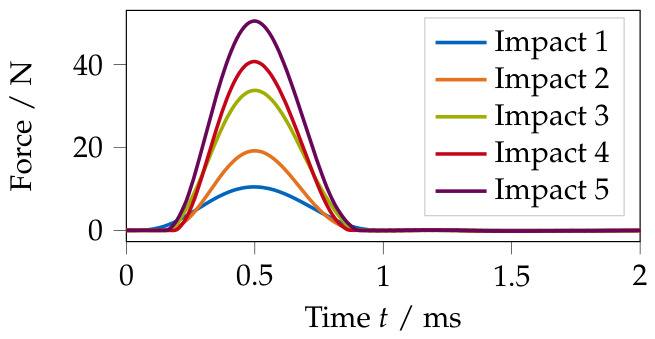
Time series of the five different applied impact forces in configuration ‘±5 g BTM Plus’.

Next, the influence of soft clipping was analyzed for frequency response functions (FRFs). Here, only results for configuration ③, with the ±5 g sensor at the bottom, are shown. Five impacts with increasing amplitude were applied, and the time series are shown in Figure 16. The force levels corresponded approximately to peak accelerations measured by the ±50 g sensor of 2, 4, 6, 8, and 10 g.

**Figure 17:**
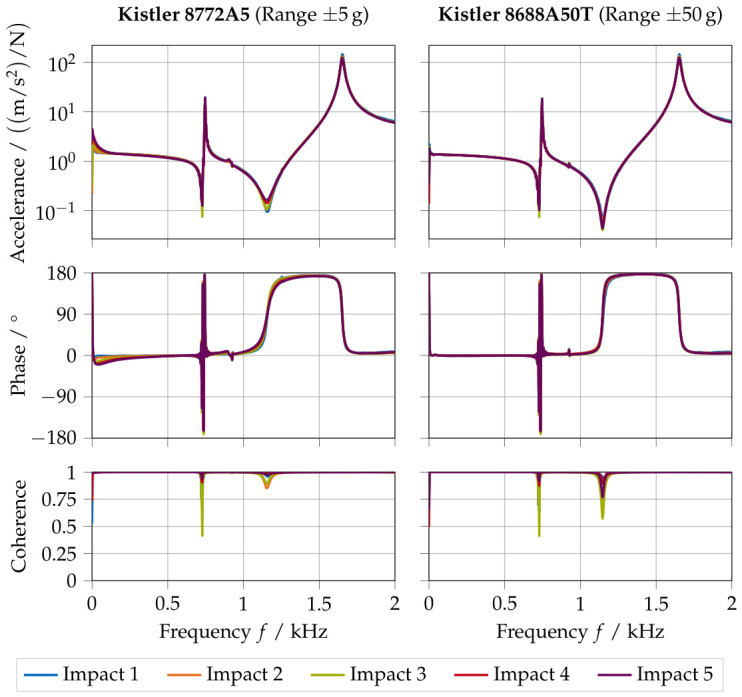
Frequency response functions for the impact test on the aluminum structure between the applied force and the respective sensor in configuration ‘±5 g BTM Plus’. The five impacts correspond approximately to peak accelerations measured by the ±50 g sensor of 2, 4, 6, 8, and 10 g.

The FRFs between the applied force and the measured response are shown in Figure 17, on the left for the ±5 g sensor and on the right for the ±50 g sensor. Due to the use of a manual impact hammer, there are slight variances between individual impacts, and thus, the anti-resonances cannot be compared between impacts.

In the resonance peaks, there is barely any difference observable, except for the right-most resonance peak; however, the difference between the impacts seems to be identical for both sensors, which rather points to a slight non-linearity of the structure than to the soft clipping effect.

The most significant difference, however, can be seen for the rigid body mode. For the ±5 g sensor, the amplitudes are increasing as the impact force increases, and, generally, the amplitudes are higher than for the ±50 g sensor. Additionally, a difference can be seen in the phase; most clearly from zero to around 500 Hz, where the phase overshoots the zero line and then slowly goes back to zero. The higher the impact force, the bigger the overshoot and the slower the return to zero. This is not visible for the ±50 g sensor. Nevertheless, a small phase overshoot is also visible for ‘Impact 2’, which did not produce accelerations that exceeded the range of the ±5 g sensor; however, the peaks are close to the limit.

This test shows that the soft clipping effect negatively affects the lower frequency range of FRFs. The increased amplitudes near 0 Hz, resulting from the introduced asymmetry during soft clipping, together with the phase drop, may serve as a good indicator of soft clipping in general. Whether soft clipping is detrimental to the measurement, again, depends on how the measurement is used further. For an experimental modal analysis that should only identify the flexible modes, the soft clipping effect might be completely irrelevant.

### 4.2. Clamped Aluminum Plate

For the second impact test, two different accelerometers from *Brüel & Kjær* were used. Both accelerometers were uniaxial and shared the same model number; however, two variants with different measurement ranges were used, where, presumably, the only difference was in the integrated electronics. After a description of the test setup, time responses and frequency response functions were compared for different impact forces, resulting in varying degrees of overload for one of the sensors.

#### 4.2.1. Test Setup

**Figure 18:**
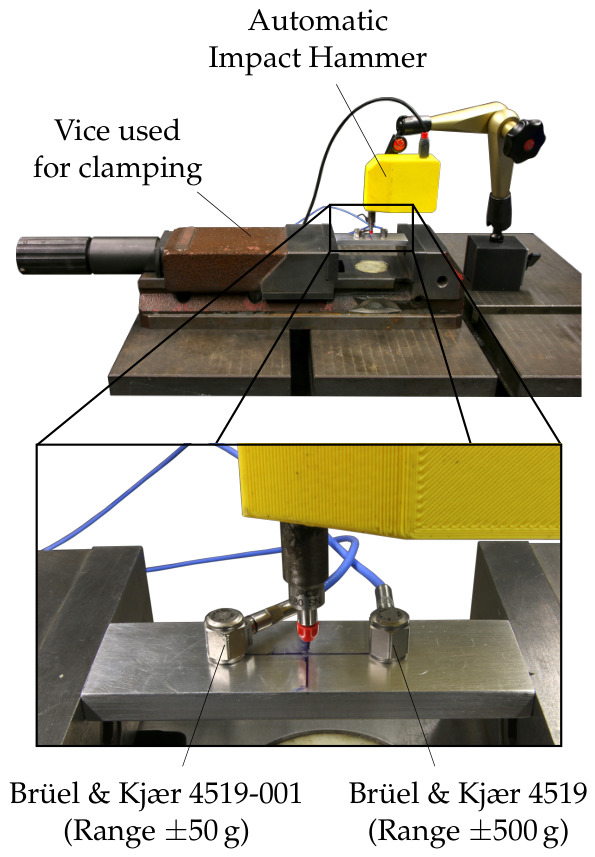
Second impact test setup with two different *Brüel & Kjær* accelerometers with acceleration ranges of ±50 g and ±500 g range.

An overview of the test setup is shown in Figure 18. Two sensors were used, a *Brüel & Kjær Type 4519-001* uniaxial accelerometer with a measurement range of ±50 g (datasheet [17]) and a *Brüel & Kjær Type 4519* uniaxial accelerometer with a measurement range of ±500 g (datasheet [18]). Using the integrated mounting stud, the sensors were screwed into a small aluminum plate, which was clamped using a vice. The plate was impacted in the middle using an automatic impact hammer. The position of the sensors and the impact was designed symmetrically, i.e., ideally, both sensors should measure the same vibrations.

**Figure 19:**
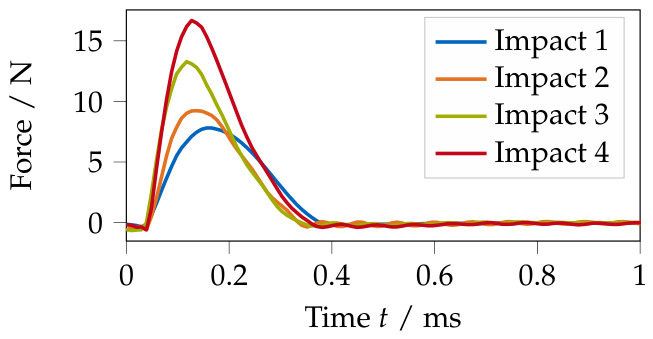
Time series of the four different impact forces applied by the auto hammer.

A *Müller-BBM PAK MKII* system was used for data acquisition with a sample rate of 102.4 kHz. With the automatic impact hammer, forces with four different amplitudes were applied; see Figure 19. From those impacts, ‘Impact 1’ and ‘Impact 2’ generated acceleration responses that were within the measurement range of the ±50 g sensor, while ‘Impact 3’ and ‘Impact 4’ exceeded the measurement range.

#### 4.2.2. Time Responses to Impacts with Varying Degree of Overload

**Figure 20:**
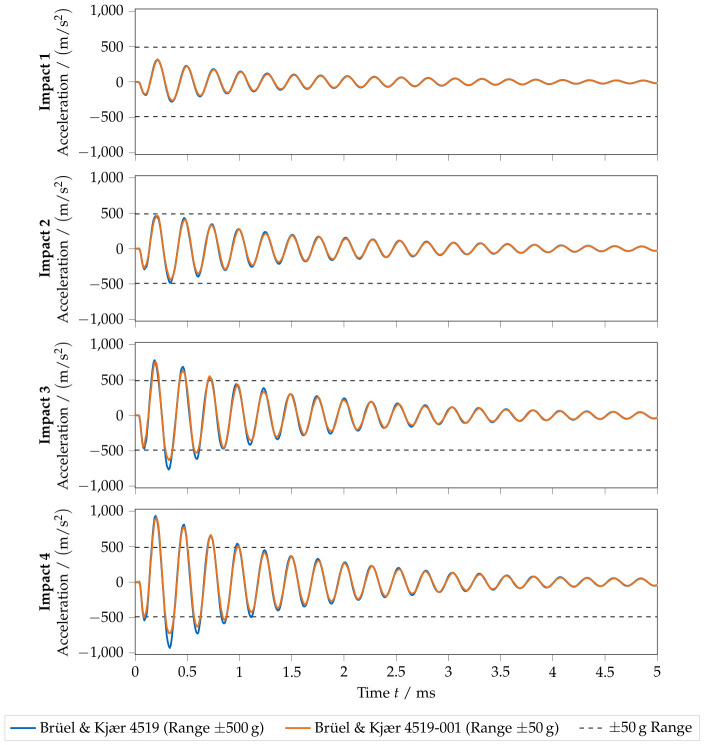
Time responses for auto impact hammer test on clamped aluminum plate. The figure shows the initial shock response over time for the *Brüel & Kjær 4519* sensor (always within measurement range) and the *Brüel & Kjær 4519-001* sensor, which is in overload outside of the ±50 g range as indicated by the dashed lines.

The acceleration time responses to the four different impacts are shown in Figure 20. As can be seen for ‘Impact 1’, with a peak acceleration of ≈33 g and being well within the measurement range of both sensors, the time response looks pretty identical, but for minor amplitude differences. For ‘Impact 2’, which is also within the measurement range, basically the same behavior can be seen; however, the negative acceleration magnitudes may already be slightly attenuated for the first few oscillation cycles. If this is really the case, is difficult to judge here, since the measured vibrations are slightly different.

For ‘Impact 3’ and ‘Impact 4’, again, a big attenuation of the negative acceleration magnitudes can be seen due to the soft clipping effect for the ±50 g sensor. This is the case for the first 2 ms, after which both sensors give the same signal again, except for some minor differences that can also be seen for ‘Impact 2’. As with the previous impact test, it can be seen that the soft clipping effect primarily affects the initial response, and as soon as the acceleration levels return to the rated measurement range, the sensor immediately behaves normally again.

#### 4.2.3. Frequency Response Functions for Varying Degree of Overload

**Figure 21:**
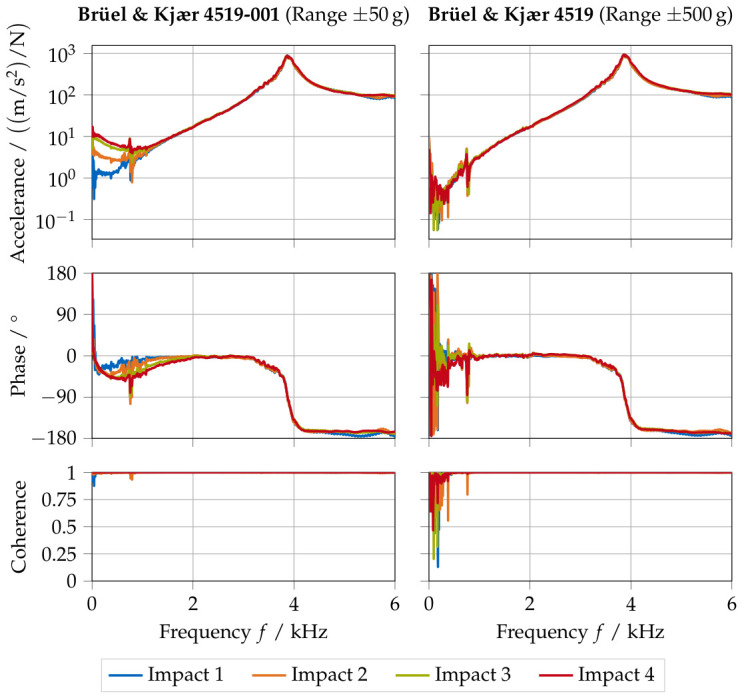
Frequency response functions for the auto impact hammer test on an aluminum plate between applied force and respective sensor. The ‘Impact 1’ and ‘Impact 2’ are within the measurement range, while ‘Impact 3’ and ‘Impact 4’ exceed the measurement range of the ±50 g sensor; see also Figure 20 for the time responses.

From the same measurements, FRFs between the applied force and both sensors can be calculated. For that, three impacts were performed for each of the four force levels and averaged. The FRFs are shown in Figure 21, on the left for the ±50 g sensor, and on the right for the ±500 g sensor.

As can be seen, the low-frequency part of the ±50 g sensor’s FRF shows higher amplitudes as the force level increases. This cannot be seen for the ±500 g sensor and is, therefore, due to the soft clipping effect. Also, the phase of the ±50 g sensor dips further below zero for higher force levels. In addition, no significant difference is observed between the two FRFs. The amplitudes of the resonance near 4 kHz do not change significantly with the force level and are practically identical for both sensors. For the frequency domain, the attenuation of the negative acceleration magnitudes does not seem that important. This can be explained by the averaging effect of the Fourier transform, where incorrect amplitudes for the initial oscillation cycles may be compensated for through subsequent cycles without attenuation.

## 5. Summary and Conclusions

This article covered the often overlooked non-linear amplitude response of IEPE sensors, referred to as soft clipping, when the rated measurement range is exceeded. The soft clipping effect is especially tricky because the rated measurement range, commonly a voltage output of ±5 V, does not coincide with the common measurement system voltage range of ±10 V. Thus, there is also no warning from the measurement system when the ±5 V range, where the sensor is specified to behave linearly, is exceeded.

For operational vibration tests, shaker tests showed that operating an IEPE sensor continuously outside the ±5 V range caused significant degradation of the measurement signal. During soft clipping, the magnitudes of the negative acceleration peaks were especially attenuated, resulting in an asymmetric signal. Furthermore, it was demonstrated that when the sensor was returned to operating conditions within its rating, a recovery time of a few seconds was required until the output had normalized again. Based on the performed measurements, the soft clipping effect appears to be frequency-independent. While the detrimental effects on the measurement were clearly visible with the fixed-frequency signals used here, in real-world operational vibrations with many different frequencies, issues due to soft clipping might be very hard to spot.

Impact tests showed that the effects of soft clipping were more subtle on shock responses. Both tests showed that mainly the initial response peaks were affected. When one is interested in the higher-frequency modes, which are quickly damped out and therefore present only in the first few oscillation cycles, the soft clipping effect could significantly impact this information. Especially for time-domain methods like Impulse-Based Substructuring (see [19] for more info), which are primarily used to accurately predict the initial shock response in the time domain, the soft clipping effect could be detrimental. For frequency-domain applications, the effect of soft clipping was found to be less relevant, except in the low-frequency range up to a couple of hundred hertz.

**Figure 22:**
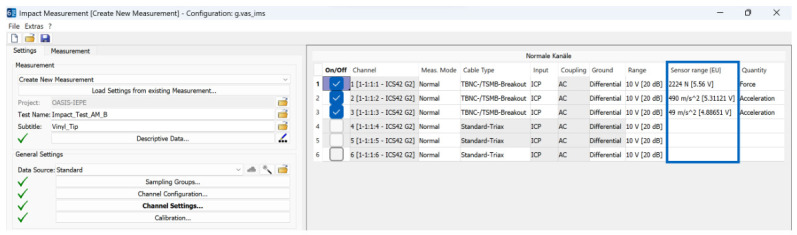
Channel setup of the *Müller-BBM PAK MKII* system with option to specify the sensor ranges in physical units (blue box).

Regarding the soft clipping effect, it is difficult to predict its impact on measurements in general. The nature of the effect, that the senor’s response slowly becomes non-linear, instead of a hard cut-off, makes it even harder to detect. Therefore, and somewhat obviously, it is best to avoid leaving the rated measurement range entirely. Nevertheless, this requires the user to either manually check each measured channel, set a voltage range for each channel within its operating range, making auto-ranging impossible, or set up a warning in the measurement system’s software. For example, for *Müller-BBM PAK MKII* systems, the user can configure the measurement ranges of the sensor using physical units in the channel setup, see Figure 22, and is then warned when this range is exceeded, instead of only when the ±10 V voltage range is exceeded.

## Data Availability

Data are available from the authors upon request.
